# Absorption and Emission Spectroscopic Investigation of the Thermal Dynamics of the Archaerhodopsin 3 Based Fluorescent Voltage Sensor Archon2

**DOI:** 10.3390/ijms21186576

**Published:** 2020-09-08

**Authors:** Alfons Penzkofer, Arita Silapetere, Peter Hegemann

**Affiliations:** 1 Fakultät für Physik, Universität Regensburg, Universitätsstraße 31, D-93053 Regensburg, Germany; 2 Experimentelle Biophysik, Institut für Biologie, Humboldt Universität zu Berlin, Invalidenstraße 42, D-10115 Berlin, Germany; arita.silapetere@hu-berlin.de (A.S.); hegemann@rz.hu-berlin.de (P.H.)

**Keywords:** Archon2, Archaerhodopsin 3, genetically encoded voltage sensor (GEVI), absorption spectroscopic characterization, fluorescence spectroscopic characterization, apparent protein melting temperature, thermal stability

## Abstract

Archon2 is a fluorescent voltage sensor derived from Archaerhodopsin 3 (Arch) of *Halorubrum sodomense* using robotic multidimensional directed evolution approach. Here we report absorption and emission spectroscopic studies of Archon2 in Tris buffer at pH 8. Absorption cross-section spectra, fluorescence quantum distributions, fluorescence quantum yields, and fluorescence excitation spectra were determined. The thermal stability of Archon2 was studied by long-time attenuation coefficient measurements at room temperature (21 ± 1 °C) and at refrigerator temperature (3 ± 1 °C). The apparent melting temperature was determined by stepwise sample heating up and cooling down (obtained apparent melting temperature: 63 ± 3 °C). In the protein melting process protonated retinal Schiff base (PRSB) with absorption maximum at 586 nm converted to de-protonated retinal Schiff base (RSB) with absorption maximum at 380 nm. Storage of Archon2 at room temperature and refrigerator temperature caused absorption coefficient decrease because of partial protein clustering to aggregates at condensation nuclei and sedimentation. At room temperature an onset of light scattering was observed after two days because of the beginning of protein unfolding. During the period of observation (18 days at 21 °C, 22 days at 3 °C) no change of retinal isomer composition was observed indicating a high potential energy barrier of S_0_ ground-state isomerization.

## 1. Introduction

Changes in the electrical potential across the plasma membrane of neurons are important for intercellular and intracellular signal transmission [1]. Recordings of membrane potentials from cells, in particular neurons, have been developed with various optical spectroscopic methods. For this purpose it is common to use either fluorescent voltage sensitive dyes [2,3], genetically encoded calcium indicators (GECIs) [4,5], or genetically encoded voltage indicators (GEVIs) [6,7]. GEVIs can be comprised of (i) voltage sensitive domains (VSD) consisting of four trans-membrane helices fused to fluorescent proteins [8,9,10,11,12,13], (ii) microbial rhodopsins consisting of 7 trans-membrane α-helices with covalently bound retinal isomers [6,7,10,11,12,14,15,16] used as stand-alone units [14,17], or fused to fluorescent proteins [18,19,20], and (iii) chemogenetic sensors [21,22,23,24,25,26,27].

In rhodopsin-based GEVIs a change in membrane voltage causes a change in fluorescence intensity which is directly used as the voltage indicator [17,28,29,30,31,32]. In the case of combined rhodopsin–fluorescent protein units (named electrochromic FRET GEVIs) the change of the rhodopsin absorption spectrum changes the efficiency of the Förster-type resonant energy transfer (FRET [33,34]) and thereby the efficiency of the fluorescent protein emission [18,19,32,35,36,37] (fluorescent protein acts as energy transfer donor and the rhodopsin acts as energy transfer acceptor [34], fluorescence of fluorescent protein is quenched by coulombic energy transfer to the rhodopsin retinal).

Generally, natural microbial rhodopsins exhibit low fluorescence quantum yield [38,39,40,41,42]. Archaerhodopsin 3 (Arch) from *Halorubrum sodomense* exhibits measurable voltage dependence of fluorescence efficiency and was studied as voltage indicator [17,28,29]. For improvement of fluorescence efficiency and voltage sensitivity, variants of Arch have been synthesized by gene technical methods [28,29,30,31,32,35,37,43,44,45,46]. Arch and Arch D95N were used for optical recording of action potentials in cultured rat hippocampal neurons [28] and in HEK-293 cells [29]. Arch D95N-D106E and Arch D95Q-D106E were used in rat hippocampal cells [35]. Archer1 (=Arch D95E-T99C) and Archer2 (=Arch D95E-T99C-A225M) were used in vivo as voltage sensors of *Caenorhabditis elegans* neurons [30]. QuasAr1 (=Arch P60S-T80S-D95H-D106H-F161V) and QuasAr2 (=QuasAr1 H95Q) were expressed in cultured rat neurons for all-optical electrophysiology studies [17]. The absorption and emission spectroscopic behavior and thermal stability of QuasAr1 was investigated in [44]. Its photocycle dynamics was studied in [45]. QuasAr3 (=QuasAr2 K171R) and paQuasAr3 (=QuasAr3 V59A) were applied to in vivo recording of supra- and subthreshold voltage dynamics from multiple neurons in mouse hippocampus [31]. QuasAr2 was fused to the fluorescent protein mOrange2 for improved recording of neuronal action potentials in cultured rat hippocampal neurons [18]. Archon1 (=Arch T20S-G41A-V44E-P60S-T80P-D86N-D95Q-D106H-A136T-F161V-T183I-L197I-G241Q) and Archon2 (=Arch T56P-P60S-T80P-D95H-T99S-T116I-F161V-T183I-L197I-A225C) were specially engineered toward optimal fluorescent voltage reporters in a robotic multidimensional-directed evolution approach [32]. They were applied to image spiking and millivolt-scale subthreshold and synaptic activity in acute mouse brain slices and in larval zebrafish in vivo [32]. Codon-optimized Archon1 (wArchon1) fused to the fluorescent protein EGFP in AVA interneurons were used to measure postsynaptic responses downstream of optogenetically controlled neurons in *Caenorhabditis elegans* [32]. The molecule Archon1-KGC-EGFP-K_V_2.1-motif-ER2, called SomArchon [37], enabled the routine population analysis of around 13 neurons at once in multiple brain regions (cortex, hippocampus, and striatum) of head-fixed, awake behaving mice [37].

Here a detailed study is presented of the absorption and emission spectroscopic properties and the thermal dynamics of Archon2 in pH 8 Tris buffer. Archon2 was selected because it exhibited the highest fluorescence signal in cultured neurons compared to other rhodopsin-based GEVIs [32]. The absorption coefficient spectrum, absorption cross-section spectrum, excitation wavelength-dependent fluorescence emission quantum distributions and quantum yields, and emission wavelength-dependent fluorescence excitation spectra of fresh prepared Archon2 at room temperature were determined. The thermal stability of Archon2 was studied by long-time spectroscopic studies at room temperature (21 ± 1 °C) and refrigerator temperature of 3 ± 1 °C. The apparent melting temperature of Archon2 was determined by stepwise sample heating up and subsequent cooling down. The thereby occurring deprotonation of protonated retinal Schiff base (PRSB) and protein denaturing are discussed. The presented results deliver basic information necessary for quantitative analysis of photo-excitation and photo-cycle studies of Archon2.

The amino acid sequence of Archon2 is shown in Figure S1 of the Supplementary Materials (Section S1). Some structural formulae of retinal cofactors of rhodopsins are taken from [42] and shown in Figure S2 of the Supplementary Materials (Section S2).

## 2. Results

### 2.1. Absorption and Emission Behavior of Fresh Thawed Archon2 Samples

**Figure 1:**
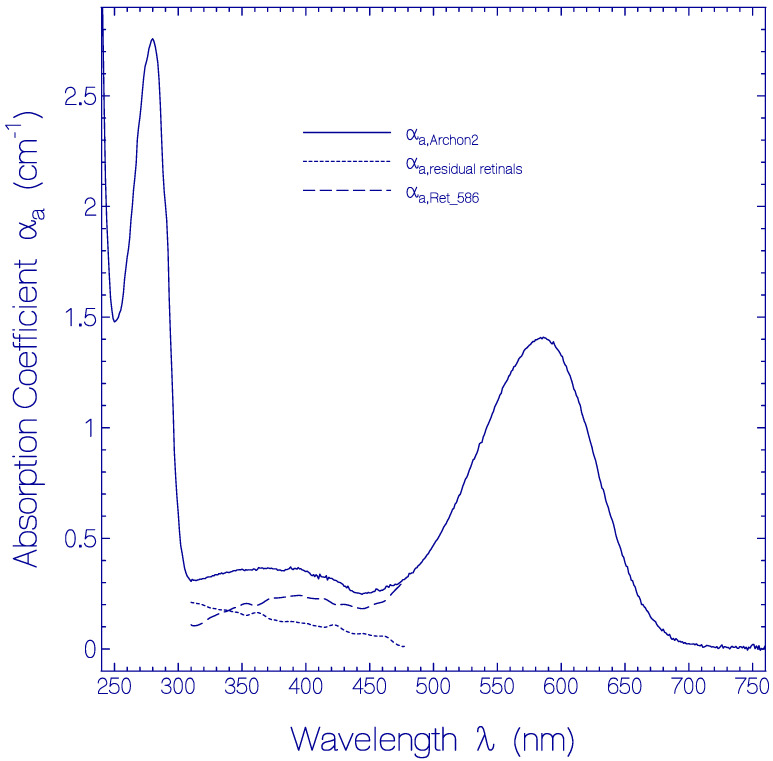
Absorption coefficient spectrum of a fresh thawed Archon2 sample in pH 8 Tris buffer at room temperature. Solid curve: measured absorption coefficient spectrum α_a,Archon2_(λ). Dashed curve: absorption coefficient spectrum α_a,Ret_586_(λ) of PRSB Ret_586. Dotted curve: absorption coefficient spectrum of residual retinal components α_a,residual retinals_(λ) = α_a,Archon2_(λ) − α_a,Ret_586_(λ).

The absorption coefficient spectrum α_a_(λ) of a fresh thawed and centrifuged Archon2 sample is shown by the solid curve in Figure 1. The main absorption band with maximum at wavelength λ = 586 nm is attributed to the S_0_–S_1_ transition of a protonated retinal Schiff base (PRSB) isomer named Ret_586. The absorption in the range from 310 nm to 480 nm is thought to be comprised of singlet S_0_–S_n_ (*n* ≥ 2) transitions of Ret_586 (dashed curve α_a,Ret_586_(λ)) and singlet ground-state–excited-state transitions of residual retinal components (dotted curve α_a,residual retinals_(λ) = α_a,Archon2_(λ) − α_a,Ret_586_(λ)). The short-wavelength absorption band peaking at λ = 280 nm is determined by apoprotein absorption (Trp, Tyr, Phe) and some retinal contribution. The α_a,Ret_586_(λ)) absorption curve in the wavelength region from 310 nm to 480 nm was determined from fluorescence excitation quantum distribution measurements presented in Figure S4 of the Supplementary Materials (Section S5).

The absorption cross-section spectrum of Ret_586, σ_a,Ret_586_(λ) = α_a,Ret_586_(λ)/*N*_Ret_586_ where *N*_Ret_586_ is the number density of Ret_586 chromophores in Archon2, is determined in Section S3 of the Supplementary Materials (Figure S3).

**Figure 2:**
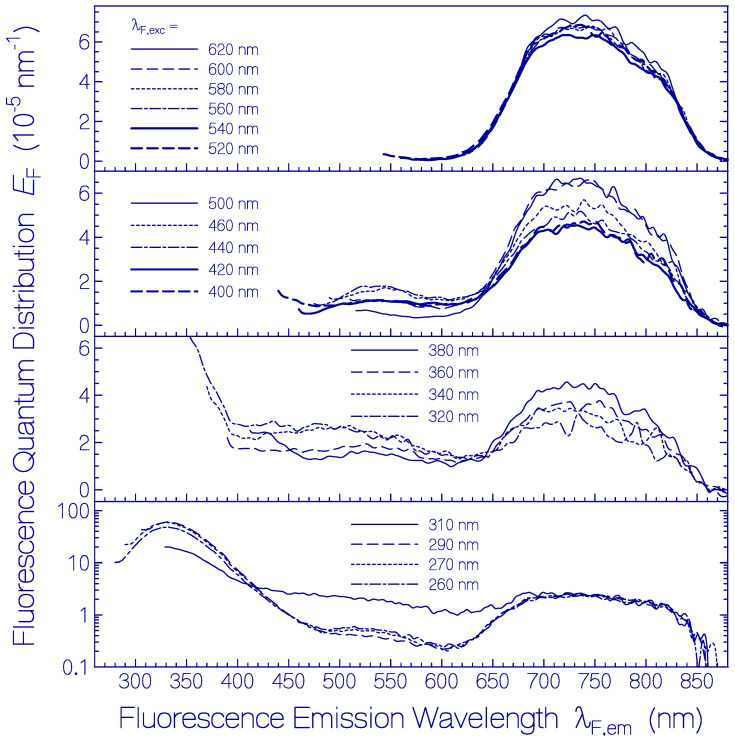
Fluorescence emission quantum distributions of fresh thawed Archon2 in pH 8 Tris buffer at room temperature. The fluorescence excitation wavelengths λ_F,exc_ are indicated in the sub-figures.

Fluorescence emission quantum distributions *E*_F_(λ) of a fresh thawed Archon2 sample in pH 8 Tris buffer are shown in Figure 2 (the same sample was used as in Figure 1). Curves are displayed for various fluorescence excitation wavelengths λ_F,exc_ in the range from 260 nm to 620 nm. For λ_F,exc_ > 520 nm only S_1_–S_0_ emission from Ret_586 is observed. The wavelength position of peak fluorescence emission is at λ_F,max_ ≈ 735 nm. The spectral half-width of this emission is $\delta {\tilde{\nu }}_{F,\mathrm{Ret}\_586}$ ≈ 2830 cm^−1^. The Stokes shift is $\delta {\tilde{\nu }}_{Stokes}=\lambda_{a,\max}^{-1}-\lambda_{F,\max}^{-1}$ ≈ 3460 cm^−1^. The strong Stokes shift and broad spectral width of Ret_586 indicate fluorescence emission along the S_1_ excited state photo-isomerization path.

Fluorescence emission in the wavelength range from λ_F,em_ = 400 nm to 600 nm is attributed to S_1_–S_0_ emission from the residual retinal isomers present in Archon2 (see dotted curve in Figure 1). The fluorescence emission band around λ_F,max_ ≈ 540 nm may be mainly due to a protonated retinal Schiff base isomer with S_0_–S_1_ absorption maximum around 470 nm. The broad fluorescence emission band in the wavelength range from λ_F,em_ ≈ 400 nm to 500 nm is attributed to deprotonated retinal Schiff base isomers in Archon2. The fluorescence emission in the range from 300 nm to 400 nm with maximum strength at λ_F,em_ ≈ 330 nm is due to apoprotein emission dominated by S_1_–S_0_ transition of Trp with absorption maximum at λ ≈ 280 nm. For all excitation wavelengths, the Ret_586 fluorescence emission band around λ_F,max_ ≈ 735 nm is present because the Ret_586 absorption extends over the whole applied fluorescence excitation wavelength region due to S_0_–S_n_ transitions (*n* ≥ 1) with fast S_n_–S_1_ nonradiative relaxation for *n* ≥ 2 and S_1_–S_0_ radiative emission. In the wavelength region of apoprotein absorption additionally Förster-type energy transfer [33,34] from Tyr and Trp to the retinal isomers contributes to the fluorescence emissions.

The total fluorescence quantum yield *ϕ*_F_ is obtained by integration of the fluorescence quantum distribution *E*_F_(λ) over the whole fluorescence emission wavelength region (*em*), i.e.,

(1) $$\phi_{F}=\int_{em}E_{F}(\lambda )d\lambda$$

**Figure 3:**
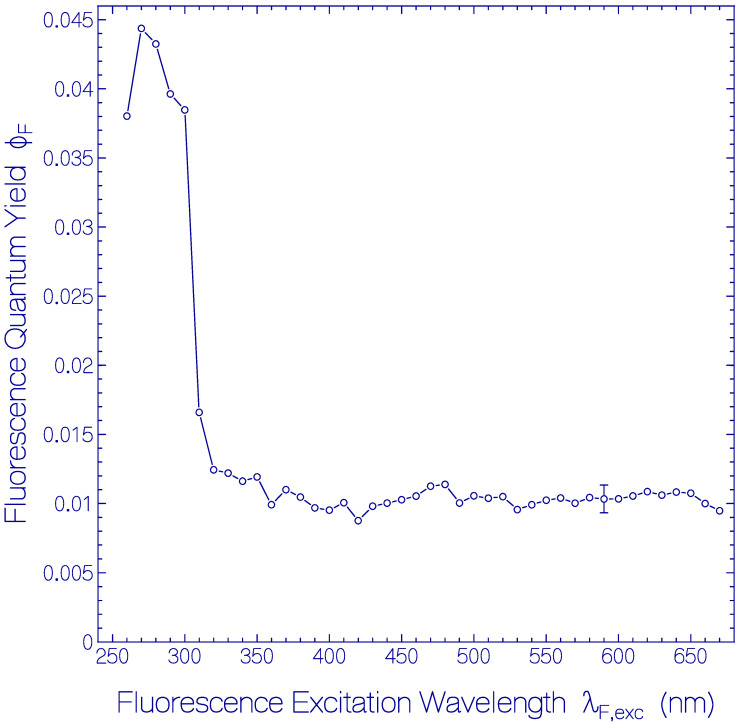
Dependence of the total fluorescence quantum yield *ϕ*_F_ on fluorescence excitation wavelength λ_F,exc_ for fresh thawed Archon2 in pH 8 Tris buffer.

The dependence of *ϕ*_F_ of the fresh thawed Archon2 sample of Figure 1 and Figure 2 on the fluorescence excitation wavelength λ_F,exc_ is shown in Figure 3. In the fluorescence excitation wavelength region from λ_F,exc_ = 320 nm to 670 nm the total fluorescence quantum yield is rather constant with a value of *ϕ*_F_ = 0.0105 ± 0.0015. This finding indicates that the fluorescence quantum yield of Ret_586 and of the additionally present retinal isomers in Archon2 have approximately the same fluorescence quantum yield. In the range from λ_F,exc_ = 260 nm to 310 nm the total fluorescence quantum yield is dominated by the apoprotein Trp emission. At λ_F,exc_ = 280 nm a fluorescence quantum yield of *ϕ*_F_ = 0.043 ± 0.003 was determined. The fluorescence emission of photo-excited Tyr is quenched by Förster-type energy transfer [33,34] to Trp (this is shown in the supplementary material of [47]). The Trp fluorescence emission is reduced by Förster-type energy transfer from Trp to the retinal isomers in Archon2. The fluorescence quantum yield of Tyr in neutral water at 20 °C is *ϕ*_F_ = 0.14 [48] and that of Trp in neutral water at 20 °C is *ϕ*_F_ = 0.15 [49,50].

The molar fluorescence brightness [51] characterizes the combined absorption and emission strength of a fluorophore. It is defined by

(2) $$B(\lambda )=\varepsilon_{a}(\lambda )\phi_{F}$$

where ε_a_(λ) is the molar decadic extinction coefficient at wavelength λ and *ϕ*_F_ is the fluorescence quantum yield. The molar decadic extinction coefficient ε_a_ is related to the molecule absorption cross-section σ_a_ by the relation

(3) $$\varepsilon_{a}(\lambda )=\frac{\sigma_{a}(\lambda )N_{A}}{\ln(10)\times 1000}$$

where *N*_A_ = 6.0221367 × 10^23^ mol^−1^ is the Avogadro constant. The maximum molar fluorescence brightness of Ret_586 is calculated to be *B*(λ_a,max_ = 586 nm) = 408 M^−1^cm^−1^ using σ_a_(586 nm) = 1.485 × 10^−16^ cm^2^ (see Figure S3) which gives ε_a_(586 nm) = 3.884 × 10^4^ M^−1^cm^−1^ (see Equation (3)), and *ϕ*_F_ = 0.0105.

In Section S4 of the Supplementary Materials the radiative lifetime τ_rad_ and the Strickler-Berg-based fluorescence lifetime τ_F,SB_ of Ret_586 are extracted from the S_0_–S_1_ absorption cross-section spectrum (Figure S3) and the fluorescence quantum yield (*ϕ*_F_(λ_F,exc_ = 580 nm) of Figure 3). The obtained results are τ_rad_(S_1_–S_0_, Ret_586) ≈ 10.1 ns and τ_F,SB_(S_1_–S_0_, Ret_586) ≈ 106 ps.

Normalized fluorescence excitation quantum distributions ${E^{\prime }}_{ex,\lambda_{F,\det}}(\lambda )$ of the fresh thawed Archon2 sample of Figure 1 and Figure 2 for fluorescence detection wavelengths λ_F,det_ in the range from 720 nm to 300 nm are shown in Figure S4 of the Supplementary Materials (Section S5). They agree with the excitation wavelength-dependent fluorescence emission quantum distributions of the dominant retinal component Ret_586 and of the other present residual retinal isomers.

### 2.2. Heating-Cooling Cycle of a Fresh Thawed Archon2 Sample

A fresh thawed sample of Archon2 in pH 8 Tris buffer was stepwise heated and then cooled. Thereby attenuation coefficient spectra α(λ) were measured. The apparent Archon2 protein melting temperature ϑ_m_ was determined by the onset of a steep attenuation rise in the transparency region of Archon2 [52] due to coalescing of denatured unfolded proteins [53]. The apparent protein melting temperature is an indicator of the protein thermal stability (the higher ϑ_m_ the more thermally stable is the protein).

**Figure 4:**
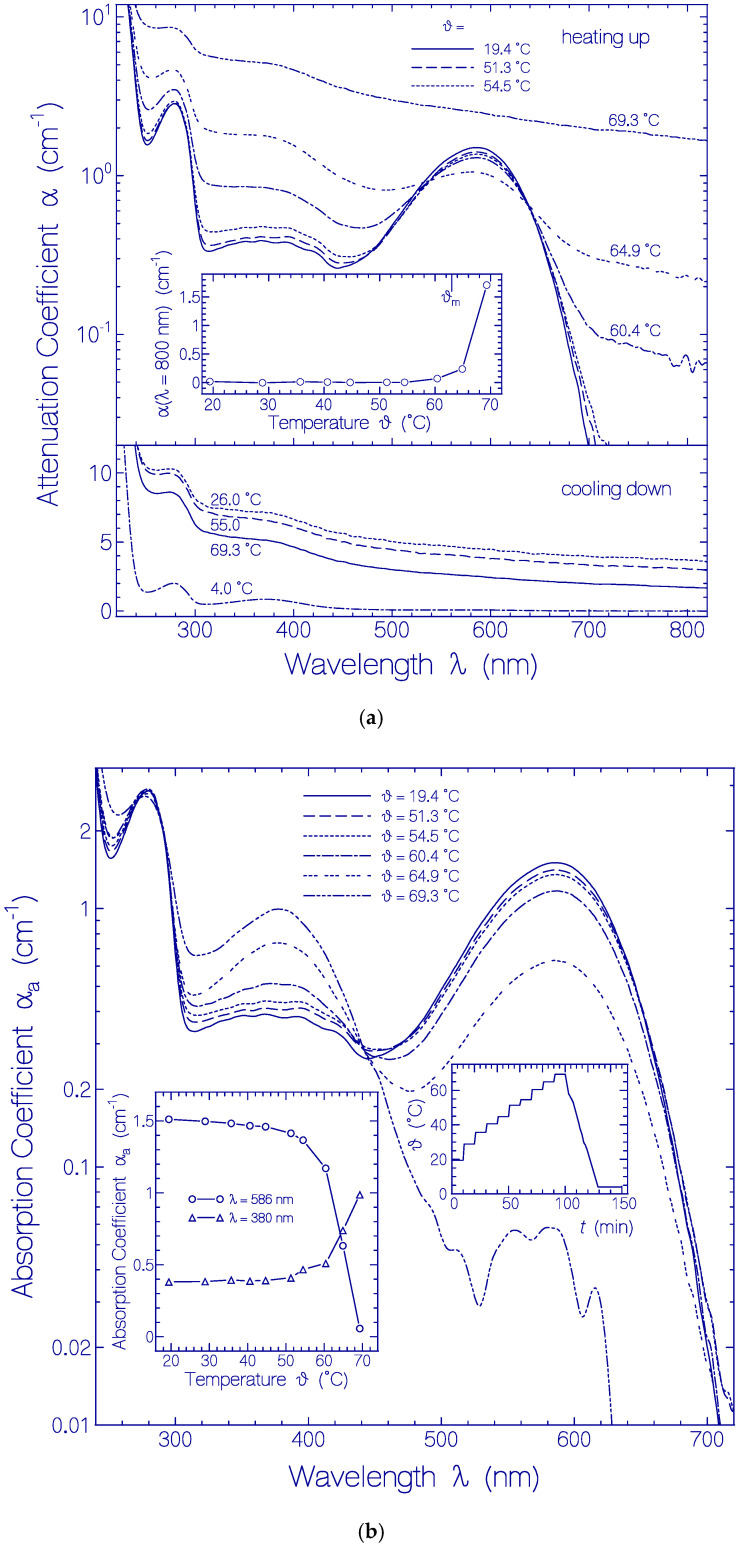
Heating-cooling cycle behavior of a fresh thawed Archon2 sample in pH 8 Tris buffer. (**a**) Attenuation coefficient spectra α(λ) development during stepwise sample heating up (top part) and cooling down (bottom part). Inset in top part: temperature-dependent attenuation coefficient development α(800 nm) during sample heating up. ϑ_m_ indicates apparent melting temperature position. (**b**) Absorption coefficient spectra α_a_(λ) development during stepwise sample heating up. Left inset: temperature-dependent absorption coefficient development α_a_(586 nm) and α_a_(380 nm). Right inset: applied heating and cooling temperature profile ϑ(*t*).

The temperature-dependent development of the attenuation coefficient spectra α(λ) of Archon2 is shown in Figure 4a. Up to 54.5 °C the attenuation spectra were nearly unchanged (see top part in Figure 4a). At 60.4 °C and 64.9 °C the attenuation spectrum around 586 nm (Ret_586 absorption) decreased and the attenuation spectrum around 380 nm (Ret_380) increased. In the Archon2 transparency region (λ > 700 nm) the light attenuation grew up because of increasing light scattering. At 69.3 °C the attenuation spectrum was strongly increased over the whole depicted wavelength region because of severe light scattering caused by Archon2 protein unfolding and clustering [53]. The temperature dependence of the light attenuation at 800 nm during the sample heating up is shown in the inset of the top part of Figure 4a. The apparent Archon2 protein melting temperature is defined by the onset of steeply rising light attenuation (light scattering). The determined value is ϑ_m_ = 63 ± 3 °C. The light scattering increased during heating up to 69.3 °C, and continued to increase during cooling down to 26 °C (see bottom part of Figure 4a). The final dash-dotted attenuation curve in the bottom part of Figure 4a was obtained after centrifugation of the sample for 30 min with 4400 rpm at 4 °C.

The temperature-dependent development of the absorption spectra α_a_(λ) of Archon2 is shown in Figure 4b. The curves were obtained from the attenuation curves shown in Figure 4a by subtracting their scattering contribution (see procedure described in Section 4.2). The PRSB Ret_586 band peaking at 586 nm decreased with rising temperature by deprotonation to RSB Ret_380 forming a rising absorption band around 380 nm. For ϑ = 69.3 °C Ret_586 is nearly fully converted to Ret_380 (absorption reduction at 586 nm by a factor of 30). Therefore the curve α_a_(λ, ϑ = 69.3 °C) in the wavelength range from 310 nm to 500 nm roughly represents the absorption coefficient spectrum of Ret_380. The absorption cross-section spectrum of Ret_380 is determined in Section S3 of the Supplementary Materials from an analysis of α_a_(λ, ϑ = 64.9 °C) (dashed curve in Figure S3).

The left inset in Figure 4b displays the temperature-dependent development of the absorption coefficients α_a_(ϑ) at λ = 586 nm (line-connected circles) and λ = 380 nm (line-connected triangles). The curves show an increasing conversion of Ret_586 to Ret_380 above ϑ = 50 °C. Below 50 °C the absorption at 380 nm is determined by the S_0_–S_n_ absorption of Ret_586 and the absorption of the present residual retinal isomers of the fresh thawed unheated Archon2 sample. The conversion of Ret_580 to Ret_380 starts already at about ϑ = 50 °C well below the apparent protein melting temperature of ϑ_m_ ≈ 63 °C.

### 2.3. Temporal Development of Archon2 at Room Temperature

The thermal stability of Archon2 in pH 8 Tris buffer at room temperature (ϑ = 21 ± 1 °C) in the dark was studied by carrying out transmission spectra measurements *T*(λ) over a duration of 18 days.

The obtained attenuation coefficient spectra α(λ) = −ln[*T*(λ)]/*l*, where *l* is the sample length, are presented in Figure S5 of the Supplementary Materials (Section S6). The attenuation coefficient spectra α(λ) are composed of absorption coefficient contributions α_a_(λ) and scattering coefficient contributions α_s_(λ). In Figure S5, onset of light attenuation in the Archon2 transparency region (λ > 700 nm) due to light scattering was observed after two days of sample storage indicating the onset of some protein unfolding with associated aggregation [53] (see discussion in Section S6 of the Supplementary Materials).

**Figure 5:**
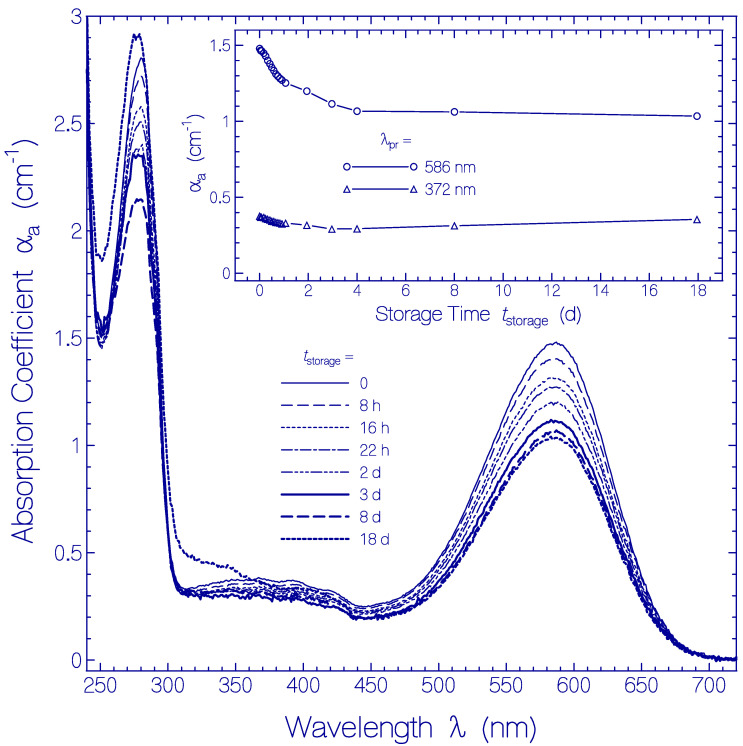
Temporal development of absorption coefficient spectra α_a_(λ,*t*_storage_) of Archon2 in pH 8 Tris buffer stored in the dark at room temperature (ϑ = 21 ± 1 °C). The storage times are listed in the legend (*t*_storage_ = 0 shows the absorption coefficient spectrum immediately after sample thawing and centrifugation). The inset shows the temporal development of α_a_(586 nm) (S_0_–S_1_ absorption of Ret_586) and of α_a_(372 nm) (S_0_–S_n_ absorption of Ret_586 and absorption of residual retinal isomers present in Archon2).

In Figure 5 the temporal development of the absorption coefficient spectra α_a_(λ) is shown. They are obtained from Figure S5 by subtracting the scattering contributions α_s_(λ) from the attenuation coefficient spectra (for procedure see below Section 4.2). The main part of Figure 5 shows absorption coefficient spectra for the storage times *t*_storage_ listed in the legend. The inset shows the development of α_a_(586 nm) and α_a_(372 nm) versus storage time. The absorption coefficient spectra decrease with storage time over the displayed wavelength range. The decrease is stronger during the first two days of storage and levels off after four days of storage. The absorption coefficient decrease with storage time is thought to be due to partial protein clustering to aggregates at condensation nuclei (absorption relevant cross-sectional area reduction of aggregates compared to monomers [54]) and subsequent onset of cluster sedimentation (sedimented clusters are no longer in the light transmission path). The absorption coefficient spectrum belonging to *t*_storage_ = 18 d shows increased absorption in the wavelength range λ < 350 nm, indicating some Archon2 apoprotein degradation.

### 2.4. Temporal Development of Archon2 at Refrigerator Temperature

The thermal stability of Archon2 in pH 8 Tris buffer at ϑ = 3 ± 1 °C in the dark was studied by carrying out frequent transmission spectra measurements during the first 22 days after sample thawing, and a final transmission spectrum measurement after 236 days.

**Figure 6:**
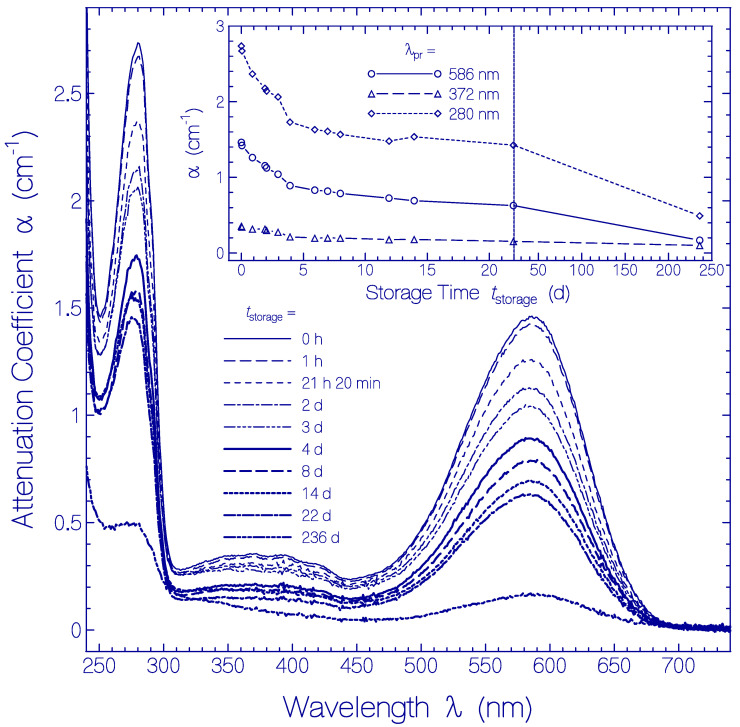
Temporal development of attenuation coefficient spectra α(λ,*t*_storage_) of Archon2 in pH 8 Tris buffer stored in the dark at temperature ϑ = 3 ± 1 °C. The storage times are listed in the legend (*t*_storage_ = 0 shows the absorption coefficient spectrum immediately after sample thawing and centrifugation). The inset shows the temporal development of α(586 nm) (S_0_–S_1_ absorption of Ret_586), of α(372 nm) (S_0_–S_n_ absorption of Ret_586 and absorption of residual retinal isomers present in Archon2), and α(280 nm) (dominant Trp absorption of apoprotein).

The temporal development of the attenuation coefficient spectra α(λ) is displayed in Figure 6. In the main part attenuation coefficient spectra are shown at selected storage times *t*_storage_ in the region between *t*_storage_ = 0 (measurement immediately after sample thawing and centrifugation) and *t*_storage_ = 236 d. The inset shows the temporal attenuation coefficient development for the selected wavelengths λ_pr_ = 586 nm (peak absorption wavelength of Ret_586), 372 nm (S_0_–S_n_ absorption of Ret_586 and absorption of residual retinal isomers) and 280 nm (wavelength position of peak apoprotein Trp absorption).

Over the whole studied storage time range no measurable scattering coefficient contribution is resolved (α_s_(λ) ≈ 0). This fact is seen in the Archon2 transparency region of λ > 700 nm, where α(λ) = α_s_(λ) ≈ 0. There is no indication of any partial protein unfolding during the investigated storage time of 236 days.

The attenuation coefficient spectra decrease with storage time is attributed to partial protein clustering to aggregates at condensation nuclei (these clusters have less effective absorption [54]) and subsequent cluster sedimentation (clusters which sedimented are no longer in the light transmission path and therefore do not contribute to absorption).

## 3. Discussion

In Section 2 we reported spectroscopic investigation of Archon2 at pH 8 in Tris buffer. The samples were studied under different conditions: (i) fresh thawed samples, (ii) thermally aged samples at refrigerator temperature (≈3 °C) and room temperature (≈21 °C), and (iii) heat-denaturized samples. The measurements provided information on the original retinal isomer composition of Archon2, the thermal stability of Archon2 at room temperature and refrigerator temperature, and the protonated retinal Schiff base (PRSB) deprotonation to neutral retinal Schiff base (RSB) in the case of thermal protein denaturation (protein melting).

### 3.1. Behavior of Fresh Thawed Archon2

In the heterologous expression of Archon2 the retinal cofactor is covalently bound to the opsin protein via a lysine Schiff base. It is dominantly present in protonated form. About 78.4% of retinal was found to be present as the protonated retinal Schiff base (PRSB) isomer Ret_586 with S_0_-S_1_ absorption maximum at 586 nm, and about 21.6% were found to be present as other retinal Schiff base isomers with S_0_–S_1_ absorption maxima below 480 nm (named residual retinals). Below 400 nm they are thought to be neutral retinal Schiff base (RSB) isomers (see Figure 1).

The absorption cross-section spectrum σ_a_(λ) of Ret_586 could be determined in Section S3 of the Supplementary Materials by determining the Archon2 concentration from the apoprotein absorption in the UV spectral range, the absorption coefficient of Archon2 at λ_a,max_ = 586 nm (there only Ret_586 is absorbing), and the fluorescence excitation spectrum *E*_ex_(λ) of Archon2 for λ_F,det_ = 700 nm (there spectral shape of *E*_ex_(λ) of Archon2 agrees with the spectral shape of σ_a_(λ) of Ret_586 in the wavelength range of λ > 310 nm).

The fluorescence quantum yield *ϕ*_F_ of the protonated retinal Schiff base isomer Ret_586 was determined to be *ϕ*_F_(S_1_–S_0_) = 0.0105 ± 0.0015. This value is large for retinals in rhodopsins where the fluorescence emission is severely quenched by photoisomerization [38,39,40,41,42]. The special engineered Archon2 variant of Arch for optimal fluorescent voltage sensing slows down the excited state isomerization dynamics which results in the desired increase of fluorescence emission efficiency.

### 3.2. Heat Denaturation of Archon2

In the stepwise Archon2 heating up and subsequent cooling down the apparent protein melting temperature ϑ_m_ of Archon2 was determined. The obtained value of ϑ_m_ ≈ 63 °C indicates high thermal protein stability. The heat denaturation of Archon2 was irreversible: after protein melting (protein unfolding) no native protein restructuring occurred in cooling down of the sample (no protein refolding). The denaturation of Archon2 resulted in deprotonation of the PRSB Ret_586 to the neutral RSB Ret_380. The protein unfolding changed the amino acid arrangement around the covalently bound retinal losing the proton fixation to the Schiff base. Data analysis in Section S3 of the Supplementary Materials allowed the determination of the absorption cross-section spectrum of Ret_380. Using the absorption cross-section spectrum together with fluorescence measurements on the denatured centrifuged sample (fluorescence quantum yield *ϕ*_F_ ≈ 0.008), the S_1_–S_0_ radiative lifetime τ_rad_ and Stickler-Berg fluorescence lifetime τ_F,SB_ of the RSB isomer Ret_380 could be approximately determined, giving τ_rad_(Ret_380) ≈ 3.31 ns and τ_F,SB_ ≈ 26.5 ps.

### 3.3. Thermal Aging of Archon2

At refrigerator temperature (≈3 °C) over a period of 22 days and at room temperature (≈21 °C) over a period of 18 days no thermal conversion of Ret_586 to other PRSB isomers and no deprotonation of Ret_586 were observed. At room temperature and lower temperature no thermal-activated S_0_ ground-state protonated retinal Schiff base isomerization and deprotonation occurred. Obviously the potential energy barrier along the S_0_ ground-state isomerization path is too high for thermal overcoming. In Section S7 of the Supplementary Materials a lower limit of the Arrhenius-type activation energy barrier *E*_act,Ret_586_ is determined to be 9152 cm^−1^ × *hc*_0_.

At refrigerator temperature over a period of 236 days no measurable attenuation coefficient increase due to light scattering was observed giving no indication of any protein denaturation (protein unfolding) during this time period at 3 °C. Over the whole wavelength region (240 nm to 680 nm) the light absorption decreased with storage time. This absorption decrease is thought to be due to partial protein clustering to aggregates at condensation nuclei and subsequent cluster sedimentation. The effective sample absorption reduces with growing cluster size because of reduced number density of non-clustered Archon2 and less effective absorption of Archon2 in clusters [54]. Sedimented clusters do not contribute to the light absorption since they are no longer in the light transmission path.

At room temperature an attenuation coefficient increase due to light scattering was seen after two days of sample storage in the dark indicating an onset of partial protein denaturation (protein unfolding, see dotted line connected square curve in the inset of Figure S5 of Section S6 of the Supplementary Materials). The absorption coefficient spectra decreased during the first four days of sample storage in the dark and then leveled off (see Figure 5). The Archon2 partial protein clustering to aggregates at condensation nuclei during the first two days at room temperature was similarly effective as at 3 °C (see insets in Figure 5 and Figure 6). But the efficiency of Archon2 partial protein clustering reduced with the onset of partial protein denaturation and leveled off after four days of storage because of continued protein unfolding.

### 3.4. Comparison of Archon2 with QuasAr1

In a previous study the thermal dynamics was investigated of the fluorescent voltage sensor QuasAr1 obtained from Archaerhodopsin 3 (Arch) by direct evolution site directed mutation [44]. Here the thermal dynamics is investigated of the fluorescent voltage sensor Archon2 derived from Arch by a robotic multidimensional-directed evolution approach for optimal fluorescent voltage sensing [32]. In the following the dark state behaviors of Archon2 and QuasAr1 are compared. Relevant parameters are collected in Table 1.

The applied molar protein concentrations were *C*(Archon2) = 20.1 μM and *C*(QuasAr1) = 27.6 μM ($C=N\times 1000/N_{A}$, *N*: number density in cm^−3^, *N*_A_: Avogadro constant in mol^−1^, *C*: molar concentration in mol dm^−3^). The absorption maximum of the dominant protonated retinal Schiff base isomer Ret_586 of Archon2 occurs at slightly longer wavelength than that of the dominant protonated retinal Schiff base isomer Ret_580 of QuasAr1 (λ_a,max,Archon2_ = 586 nm, λ_a,max,QuasAr1_ = 580 nm). The amount of residual retinal isomers besides the dominant PRSB is higher in Archon2 than in QuasAr1 (κ_residual retinals,Archon2_ ≈ 0.218, κ_residual retinals,QuasAr1_ ≈ 0.138). The fluorescence quantum yield of Ret_586 of Archon2 is a factor of 1.6 larger than the fluorescence quantum yield of Ret_580 of QuasAr1 (*ϕ*_F,Ret_586 of Archon2_ ≈ 0.0105, *ϕ*_F,Ret_580 of QuasAr1_ ≈ 0.0065). The apparent melting temperature of Archon2 is slightly lower than that of QuasAr1 (ϑ_m,Archon2_ ≈ 63 °C, ϑ_m,QuasAr1_ ≈ 65 °C). The similarity of the apparent melting temperatures indicates similar thermal stability of Archon2 and QuasAr1.

The thermal development of Archon2 and QuasAr1 versus storage time in the dark at room temperature is different. Onset of light scattering, which is an indication of the onset of protein unfolding, is observed earlier for QuasAr1 than for Archon2 (*t*_onset of light scattering_ ≈ 12 h for QuasAr1 and ≈ 48 h for Archon2). The initial rate of absorption decrease due to partial protein clustering to aggregates at condensation nuclei is large for Archon2 and negligible for QuasAr1 ($\left(-\partial \alpha_{a}(\lambda_{a,\max})/\partial t_{storage}{|}_{t_{storage}=0}\right)/\alpha_{a}(\lambda_{a,\max},t_{storage}=0)$≈ 0.14 d^−1^ for Archon2 and ≈ 0 for QuasAr1). For Archon2 no ground-state isomerization of the PRSB Ret_586 isomer to other PRSB isomers and no deprotonation of Ret_586 are observed. For QuasAr1 ground-state isomerization of PRSB Ret_580 to other PRSB isomers and subsequent deprotonation occurred. Ret_580 was found to be composed of the PRSB isomers Ret_580_I_ and Ret_580_II_ whereby Ret_580_I_ converted the PRSB Ret_530, and Ret_580_II_ converted to the PRSB Ret_640. Ret_530 deprotonated to the RSB Ret_400, and Ret_640 deprotonated to the RSB Ret_350. The non-occurrence of ground-state isomerization of Archon2 is attributed to a high ground-state activation barrier (*E*_act,Ret_586_ > 9152 cm^−1^ × *hc*_0_ (see Section S7 of the Supplementary Materials), while $E_{act,{\mathrm{Ret}\_580}_{I}}$ = 8670 cm^−1^ × *hc*_0_ and $E_{act,{\mathrm{Ret}\_580}_{\mathrm{II}}}$ = 9380 cm^−1^ × *hc*_0_ [44]). The non-observation of Ret_586 ground-state isomerization hinders to get any information whether Ret_586 of Archon2 is composed of an isomer mixture as was resolved for Ret_580 of QuasAr1.

At refrigerator temperature thermal protein unfolding plays no role both for Archon2 and QuasAr1 (*t*_onset of light scattering_ ≈ 50 d for QuasAr1 and > 236 d for Archon2). The initial rate of absorption decrease due to partial protein clustering to aggregates at condensation nuclei is higher for Archon2 than for QuasAr1 ($\left(-\partial \alpha_{a}(\lambda_{a,\max})/\partial t_{storage}{|}_{t_{storage}=0}\right)/\alpha_{a}(\lambda_{a,\max},t_{storage}=0)$≈ 0.15 d^−1^ for Archon2 and ≈ 0.054 d^−1^ for QuasAr1). No ground-state isomerization and no deprotonation of the PRSB Ret_586 are observed for Archon2 in the dark at 3 °C over a period of 22 days. For QuasAr1 ground-state isomerization of PRSB Ret_580 to other PRSB isomers and subsequent deprotonation was found to be small in the dark at 2.5 °C.

## 4. Materials and Methods

### 4.1. Sample Preparation

For high yield purification, an *E.coli* codon-optimized genes encoding for Arch (UniProtKB-P96787 amino acids 1–253, with mutations adapted from [32]) with a TEV protease cleavage site and a 6xHis tag at the C-terminal (ENLYFQGLEHHHHHH) were synthesized by (GenScript, Nanjing, China) and cloned into a pET21a(+) expression vector between the NdeI and SalI restriction sites. In difference to Arch the following mutants were introduced: T56P-P60S-T80P-D95H-T99S-T116I-F161V-T183I-L197I-A225C.

The expression plasmid (pET21a(+)) carrying Archon2 was transformed into C41(DE3) *E. coli* cells. To induce the protein expression we used 0.5 mM isopropyl β-D-thiogalactopyranoside (IPTG; Carl Roth GmbH, Karlsruhe, Germany) and the LB media was supplemented with 5 µM all-*trans* retinal (ATR; Sigma-Aldrich, St. Louis, MO, USA). The cells were incubated at 37 °C for 4 h and then harvested. The cells were disrupted using an EmulsiFlex-C3 Homogenizer (AVESTIN Inc., Ottawa, Canada). The membrane fraction was collected by ultracentrifugation (45,000 rpm) for 1 h at 4 °C (Type 45 Ti; Beckman Inc., Indianapolis, IN, USA) and then resuspended in buffer containing 50 mM Tris-HCl (pH 8.0), 300 mM NaCl, 0.1 mM PMSF, 1.5% *n*-dodecyl-β-d-maltoside (DDM, GLYCON Biochemicals GmbH, Luckenwalde, Germany), and 0.3% cholesteryl hemisuccinate (CHS, Sigma-Aldrich, St. Louis, MO, USA) and stirred overnight for solubilization. The insoluble fraction was removed by ultracentrifugation (200,000× *g*, 1 h at 4 °C). The Archon2 protein was purified by Ni-NTA affinity and using an ÄKTAxpress protein purification system (GE Healthcare Life Science, Chicago, IL, USA) configured with a HisTrap HP Ni-NTA column. The protein was collected in the final buffer containing 50 mM Tris-HCl (pH 8.0), 150 mM NaCl, 0.1 mM PMSF, 0.02% DDM, and 0.004% CHS. The purity of the protein was checked by a SDS-PAGE gel investigation. The expressed Archon2 solution was aliquoted to amounts of 30 μL in Eppendorf tubes, shock-frozen, and stored at −80 °C.

### 4.2. Spectroscopic Measurements

For experimental investigations the Archon2 samples stored at −80 °C were thawed and then transferred to ultra-micro fused silica fluorescence cells (inner cell dimensions relevant for measurements: 3 mm length, 1.5 mm width, and 5 mm height; special fabrication of Hellma GmbH, Müllheim/Baden, Germany). Fresh thawed samples were centrifuged with 4400 rpm for 30 min at 4 °C (Centrifuge 5702 R, Eppendorf AG, Hamburg, Germany).

Transmission measurements versus wavelength, *T*(λ), were carried out with a spectrophotometer (Cary 50, Varian Australia Pty Ltd., Mulgrave, Victoria, Australia). Attenuation coefficient spectra were calculated by the relation, α(λ)=−ln[T(λ)]/l, were *l* is the sample length. In the case of negligible protein light scattering the attenuation coefficient spectrum α(λ) is equal to the absorption coefficient spectrum α_a_(λ). The attenuation coefficient spectrum α(λ) comprises absorption (α_a_) and scattering (α_s_) contributions according to $\alpha (\lambda )=\alpha_{a}(\lambda )+\alpha_{s}(\lambda )$. The scattering coefficient spectrum is approximated by the empirical relation [55] $\alpha_{s}(\lambda )=\alpha_{s}(\lambda_{0}){\left(\lambda_{0}/\lambda \right)}^{\gamma }$ where the wavelength λ_0_ is selected in the transparency region and γ ≤ 4 is fitted to the experimental attenuation in the transparency region. Absorption coefficient spectra were determined by subtracting the scattering contribution from the measured attenuation coefficient spectra.

The Archon2 melting was studied by stepwise sample heating up, then cooling down and thereby measuring the attenuation coefficient spectra development [52,53]. The apparent protein melting temperature ϑ_m_ was derived from the onset of strong light attenuation in the transparency region of Archon2.

The thermal stability of Archon2 at room temperature (21 ± 1 °C) and refrigerator temperature (3 ± 1 °C) was determined by storing Archon2 samples at these temperatures in the dark and measuring transmission spectra at certain time intervals.

Fluorescence spectroscopic measurements were carried out with a spectrofluorimeter (Cary Eclipse, Varian Australia Pty Ltd., Mulgrave, Victoria, Australia). Fluorescence emission quantum distributions *E*_F_(λ) were determined from fluorescence emission spectra measurements at fixed excitation wavelengths [33,56,57]. The dye rhodamine 6G in methanol was used as reference standard for fluorescence quantum distribution calibration (fluorescence quantum yield *ϕ*_F,ref_ = 0.93 [58]). The fluorescence quantum yield *ϕ*_F_ was calculated using the relation $\phi_{F}=\int_{em}E_{F}(\lambda )d\lambda$ where the integration runs over the fluorescence emission wavelength region (see Equation (1) above). Fluorescence excitation quantum distributions *E*_ex_(λ) were recorded by scanning the fluorescence excitation wavelength over the absorption wavelength region at fixed fluorescence detection wavelengths λ_F,det_ [59]. Magic angle conditions were applied for the fluorescence recordings (vertical polarized excitation and orientation of the fluorescence detection polarizer at an angle of 54.7° to the vertical [60]). The spectra were corrected for the spectral sensitivity of the spectrometer and the photodetector.

## 5. Conclusions

The Archaerhodopsin 3-based fluorescent voltage sensor Archon2 optimized by a robotic multidimensional directed evolution approach [32] was characterized by its absorption and emission spectroscopic behavior and its long-time thermal stability. The measured fluorescence quantum yield of *ϕ*_F_ ≈ 1.05% was approximately a factor of 1.6 higher than that of the previously highest fluorescent Archaerhodopsin 3-based voltage sensor QuasAr1 [17,44]. Archon2 in pH 8 Tris buffer turned out to be thermally very stable in the dark. At room temperature after two days of storage in the dark some onset of light scattering was observed indicating an onset of partial protein unfolding. The attenuation spectrum decreased with storage time before the onset of light scattering indicating partial native protein clustering to aggregates at condensation nuclei before the onset of partial protein unfolding. At refrigerator temperature no protein unfolding occurred during long-time storage in the dark, only partial protein clustering at condensation nuclei and cluster sedimentation were observed. At refrigerator temperature and even room temperature the Archon2 protonated retinal Schiff base isomer composition and deprotonated retinal Schiff base isomer composition did not change with storage time indicating high activation barriers for ground-state isomerization and protonation/deprotonation changes.

## Figures and Tables

**Table 1:** Comparison of thermal behavior of Archon2 with QuasAr1.

Parameter	Archon2	QuasAr1
*C* (mol dm^−3^)	2.01 × 10^−5^	2.76 × 10^−5^
**Fresh Thawed Sample**		
Dominant PRSB	Ret_586	Ret_580
λ_a,max_ (nm)	586	580
κ	0.784	0.862
σ_a_(λ_a,max_) (cm^2^)	1.485 × 10^−16^	1.593 × 10^−16^
σ_a,int_ (cm)	5.75 × 10^−13^	6.1 × 10^−13^
*f*	0.546	0.58
*ϕ*_F_	(1.05 ± 0.15) × 10^−2^	(6.5 ± 0.5) × 10^−3^
*B*_max_ (M^−1^cm^−1^)	408 ± 60	270 ± 20
τ_rad_ (ns)	10.1	9.32
τ_F,SB_ (ps)	106	61.5
**Heat Denatured Sample**		
ϑ_m_ (°C)	63 ± 3	65 ± 3
Dominant RSB	Ret_380	Ret_380
λ_a,max_ (nm)	380	380
σ_a_(λ_a,max_) (cm^2^)	9.08 × 10^−17^	7.4 × 10^−17^
σ_a,int_ (cm)	5.95 × 10^−13^	5.2 × 10^−13^
*f*	0.56	0.49
*ϕ*_F_	0.008 ± 0.001	≈0.04
τ_rad_ (ns)	3.31	3.74
τ_F_ (ps)	26.5	150
**Aging at Room Temperature**		
ϑ (°C)	21 ± 1	21–25
*t*_onset of light scattering_ (h)	≈48	≈12
$\frac{-\partial \alpha_{a}(\lambda_{a,\max})/\partial t_{storage}{|}_{t_{storage}=0}}{\alpha_{a}(\lambda_{a,\max},t_{storage}=0)}$ (d^−1^)	≈0.14	≈0
Ground-state isomerization	no	yes
**Aging at Refrigerator Temperature**		
ϑ (°C)	3 ± 1	2.5 ± 0.5
*t*_onset of light scattering_ (d)	>22	≈50
$\frac{-\partial \alpha_{a}(\lambda_{a,\max})/\partial t_{storage}{|}_{t_{storage}=0}}{\alpha_{a}(\lambda_{a,\max},t_{storage}=0)}$ (d^−1^)	≈0.15	0.054
Ground-state isomerization	no	small

Abbreviations: *C*: applied protein molar concentration of fresh thawed sample. λ_a,max_: wavelength position of maximum absorption cross-section of the S_0_–S_1_ absorption band of the considered retinal Schiff base isomer. κ: fraction of protonated retinal Schiff base. σ_a_(λ_a,max_): absorption cross-section at λ_a,max_. σ_a,int_: absorption cross-section integral of considered S_0_-S_1_ absorption band. *f*: oscillator strength. *ϕ*_F_: fluorescence quantum yield. *B*_max_: maximum molar fluorescence brightness. τ_rad_: radiative lifetime. τ_F,SB_: Strickler-Berg fluorescence lifetime. ϑ: temperature. *t*_onset of light scattering_: time of sample storage in the dark before observation of light scattering. $\left(-\partial \alpha_{a}(\lambda_{a,\max})/\partial t_{storage}{|}_{t_{storage}=0}\right)/\alpha_{a}(\lambda_{a,\max},t_{storage}=0)$: initial rate of absorption decrease at λ_a,max_.

## Supplementary Materials

Supplementary Materials can be found at https://www.mdpi.com/1422-0067/21/18/6576/s1. Figure S1. Amino acid sequence of Archon2; Figure S2. Structural formulae of some protonated and neutral retinal Schiff base isomers; Figure S3. Absorption cross-section spectra of Ret_586 and Ret_380; Figure S4. Normalized fluorescence excitation quantum distributions of Archon2 in pH 8 Tris buffer; Figure S5. Temporal development of attenuation coefficient spectra of Archon2 in pH 8 Tris buffer at room temperature. References [61,62,63,64,65,66,67,68,69,70] are cited in the Supplementary Materials.

## Abbreviations

Arch	Archaerhodopsin 3 from *Halorubrum sodomense*
Archon1	Rhodopsin based on Arch with thirteen point mutations
Archon2	Rhodopsin based on Arch with ten point mutations
FRET	Förster resonance energy transfer
GECI	Genetically encoded calcium indicator
GEVI	Genetically encoded voltage indicator
PRSB	Protonated retinal Schiff base
QuasAr	Quality superior to Arch
Ret_xxx	Retinal with absorption maximum approximately at xxx nm
RSB	Retinal Schiff base
Trp	Tryptophan
Tyr	Tyrosine
VSD	Voltage sensing domain

## References

[1] Ainsworth M., Lee S., Cunningham M.O., Traub R.D., Kopell N.J., Whittington M.A. (2012). Rates and rhythms: A synergistic view of frequency and temporal coding in neural networks. Neuron.

[2] Shoham D., Glaser D.E., Arieli A., Kenet T., Wijnbergen C., Toledo Y., Hildesheim R., Grinvald A. (1999). Imaging cortical dynamics at high spatial and temporal resolution with novel blue voltage-sensitive dyes. Neuron.

[3] Ortiz G., Liu P., Naing S.H.H., Muller V.R., Miller E.W. (2019). Synthesis of sulfonated carbofluoresceins for voltage imaging. J. Am. Chem. Soc..

[4] Mank M., Griesbeck O. (2008). Genetically encoded calcium indicators. Chem. Rev..

[5] Nguyen C., Upadhyay H., Murphy M., Borja G., Rozsahegyi E.M., Barnett A., Brookings T., McManus O.B., Werley C.A. (2019). Simultanous voltage and calcium imaging and optogenetic stimulation with high sensitivity and a wide field of view. Biomed. Opt. Express.

[6] Bando Y., Grimm C., Cornejo V.H., Yuste R. (2019). Genetic voltage indicators. BMC Biol..

[7] Panzera L.C., Hoppa M.B. (2019). Genetically encoded voltage indicators are illuminating subcellular physiology of the axon. Front. Cell. Neurosci..

[8] Murata Y., Iwasaki H., Sasaki M., Inaba K., Okamura Y. (2005). Phosphoinositide phosphatase activity coupled to an intrinsic voltage sensor. Nature.

[9] Mutoh H., Akemann W., Knöpfel T. (2012). Genetically engineered fluorescent voltage reporters. ACS Chem. Neurosci..

[10] Lin M.Z., Schnitzer M.J. (2016). Genetically encoded indicators of neuronal activity. Nat. Neurosci..

[11] Xu Y., Zou P., Cohen A.E. (2017). Voltage imaging with genetically encoded indicators. Curr. Opin. Chem. Biol..

[12] Bando Y., Sakamoto M., Kim S., Ayzenshtat I., Yuste R. (2019). Comparative evaluation of genetically encoded voltage indicators. Cell Rep..

[13] Kang B.E., Lee S., Baker B.J. (2019). Optical consequences of a genetically-encoded voltage indicator with a pH sensitive fluorescent protein. Neurosci. Res..

[14] Kralj J.M., Hochbaum D.R., Douglass A.D., Cohen A.E. (2011). Electrical spiking in *Escherichia coli* probed with a fluorescent voltage-indicating protein. Science.

[15] Gong Y. (2015). The evolving capabilities of rhodopsin-based genetically encoded voltage indicators. Curr. Opin. Chem. Biol..

[16] Hashemi N.A., Beergs A.C.F., Schüler C., Scheiwe A.R., Costa W.S., Bach M., Liewald J.F., Gottschalk A. (2019). Rhodopsin-based voltage imaging tools for use in muscles and neurons of *Caenorhabditis elegans*. Proc. Natl. Acad. Sci. USA.

[17] Hochbaum D.R., Zhao Y., Farhi S.L., Klapoetke N., Werley C.A., Kapoor V., Zou P., Kralj J.M., Maclaurin D., Smedemark-Margulies N. (2014). All-optical electrophysiology in mammalian neurons using engineered microbial rhodopsins. Nat. Methods.

[18] Zou P., Zhao Y., Douglass A.D., Hochbaum D.R., Brinks D., Werley C.A., Harrison D.J., Campbell R.E., Cohen A.E. (2014). Bright and fast multicoloured voltage reporters via electrochromic FRET. Nat. Commun..

[19] Gong Y., Wagner M.J., Li Z., Schnitzer M.J. (2014). Imaging neural spiking in brain tissue using FRET-opsin protein voltage sensors. Nat. Commun..

[20] Gong Y., Huang C., Li J.Z., Grewe B.F., Zhang Y., Eismann S., Schnitzer M.J. (2015). High-speed recoding of neural spikes in awake mice and flies with a fluorescent voltage sensor. Science.

[21] González J.E., Tsien R.Y. (1995). Voltage sensing by fluorescence resonance energy transfer in single cells. Biophys. J..

[22] Chanda B., Blunck R., Faria L.C., Schweizer F.E., Mody I., Bezanilla F. (2005). A hybrid approach to measuring electrical activity in genetically specified neurons. Nat. Neurosci..

[23] Abdelfattah A.S., Farhi S.L., Zhao Y., Brinks D., Zou P., Ruangkittisakul A., Platisa J., Pieribone V.A., Ballanyi K., Cohen A.E. (2016). A bright and fast red fluorescent voltage indicator that reports neuronal activity in organotypic brain slices. J. Neurosci..

[24] Lui P., Grenier V., Hong W., Muller V.R., Miller E.W. (2017). Fluorgenic targeting of voltage-sensitive dyes to neurons. J. Am. Chem. Soc..

[25] Grenier V., Daws B.R., Liu P., Miller E.W. (2019). Spying on neuronal membrane potential with genetically targetable voltage indicators. J. Am. Chem. Soc..

[26] Sundukova M., Prifti E., Bucci A., Krillova K., Serrao J., Reymond L., Umebayashi M., Hovius R., Riezman H., Johnsson K. (2018). A chemogenetic approach for optical monitoring of voltage in neurons. Angew. Chem. Int. Ed..

[27] Abdelfattah A.S., Kawashima T., Singh A., Novak O., Liu H., Shuai Y., Huang Y.-C., Campagnola L., Seeman S.C., Yu J. (2019). Bright and photostable chemigenetic indicators for extended in vivo voltage imaging. Science.

[28] Kralj J., Douglass A.D., Hochbaum D.R., Maclaurin D., Cohen A.E. (2012). Optical recording of action potentials in mammalian neurons using a microbial rhodopsin. Nat. Methods.

[29] Maclaurin D., Venkatachalam V., Lee H., Cohen A.E. (2013). Mechanism of voltage-sensitive fluorescence in a microbial rhodopsin. Proc. Natl. Acad. Sci. USA.

[30] Flytzanis N.C., Bedbrook C.N., Engqvist M.K.M., Xiao C., Chan K.Y., Sternberg P.W., Arnold F.H., Gradinaru V. (2014). Archaerhodopsin variants with enhanced voltage-sensitive fluorescence in mammalian and *Caenorhabditis elegans* neurons. Nat. Commun..

[31] Adam Y., Kim J.J., Lou S., Zhao Y., Brinks D., Wu H., Mostajo-Radji M.A., Kheifets S., Parot V., Chettih S. (2018). All-optical electrophysiology reveals brain-state dependent changes in hippocampal subthreshold dynamics and excitability. bioRxiv.

[32] Piatkevich K.D., Jung E.E., Straub C., Linghu C., Park D., Suk H.J., Hochbaum D.R., Goodwin D., Pnevmatikakis E., Pak N. (2018). A robotic multidimensional directed evolution approach applied to fluorescent voltage reporters. Nat. Chem. Biol..

[33] Förster T. (1951). Fluoreszenz Organischer Verbindungen.

[34] Valeur B., Berberan-Santos M.N. (2012). Molecular Fluorescence: Principles and Applications.

[35] Gong Y., Li J.Z., Schnitzer M.J. (2013). Enhanced Archaerhodopsin fluorescent protein voltage indicators. PLoS ONE.

[36] Kannan M., Vasan G., Huang C., Haziza S., Li J.Z., Inan H., Schnitzer M.J., Pieribone V.A. (2018). Fast, in vivo voltage imaging using a red fluorescent indicator. Nat. Methods.

[37] Piatkevich K.D., Bensussen S., Tseng H.-A., Shroff S.N., Lopez-Huerta V.G., Park G., Jung E.E., Shemesh O.A., Straub C., Gritton H.J. (2019). Population imaging of neural activity in awake behaving mice. Nature.

[38] Kandori H., Shichida Y., Yoshizawa T. (2001). Photoisomerization in rhodopsin. Biochemistry.

[39] Wand A., Gdor I., Zhu J., Sheves M., Ruhman S. (2013). Shedding new light on retinal protein photochemistry. Annu. Rev. Phys. Chem..

[40] Alexiev U., Farrens D.L. (2014). Fluorescence spectroscopy of rhodopsins: Insights and approaches. Biochim. Biophys. Acta.

[41] Ernst O.P., Lodowski D.T., Elstner M., Hegemann P., Brown L.S., Kandori H. (2014). Microbial and animal rhodopsins: Structures, functions, and molecular mechanisms. Chem. Rev..

[42] Penzkofer A., Hegemann P., Kateriya S., Duarte F.J. (2018). Organic dyes in optogenetics. Organic Lasers and Organic Photonics.

[43] Adam Y., Kim J.J., Lou S., Zhao Y., Xie M.E., Brinks D., Wu H., Mostajo-Radji M.A., Kheifets S., Parot V. (2019). Voltage imaging and optogenetics reveal behaviour-dependent changes in hippocampal dynamics. Nature.

[44] Penzkofer A., Silapetere A., Hegemann P. (2019). Absorption and emission spectroscopic investigation of the thermal dynamics of the Archaerhodopsin 3 based fluorescent voltage sensor QuasAr1. Int. J. Mol. Sci..

[45] Penzkofer A., Silapetere A., Hegemann P. (2020). Photocycle dynamics of the Archaerhodopsin 3 based fluorescent voltage sensor QuasAr1. Int. J. Mol. Sci..

[46] Lou S., Adam Y., Weinstein E.N., Williams E., Williams K., Parot V., Kavokine N., Liberles S., Madisen L., Zeng H. (2016). Genetically targeted all-optical electrophysiology with a transgenic Cre-dependent Optopatch mouse. J. Neurosci..

[47] Penzkofer A., Luck M., Mathes T., Hegemann P. (2014). Bistable retinal Schiff base photodynamics of histidine kinase rhodopsin HKR1 from *Chlamydomonas reinhardtii*. Photochem. Photobiol..

[48] Chen R.F. (1967). Fluorescence quantum yields of tryptophan and tyrosine. Anal. Lett..

[49] Eisinger J., Navon G. (1969). Fluorescence quenching and isotope effect of tryptophan. J. Chem. Phys..

[50] Kirby E.P., Steiner R.F. (1970). The influence of solvent and temperature upon the fluorescence of indole derivatives. J. Phys. Chem..

[51] Reineck P., Francis A., Orth A., Lau D.W.M., Nixon-Luke R.D.V., Rastogi I.D., Razali W.A.W., Cordina N.M., Parker L.M., Sreenivasan V.K.A. (2016). Brightness and photostability of emerging red and near-IR fluorescent nanomaterials for bioimaging. Adv. Opt. Mater..

[52] Penzkofer A., Scheib U., Hegemann P., Stehfest K. (2016). Absorption and emission spectroscopic investigation of thermal dynamics and photo-dynamics of the rhodopsin domain of the rhodopsin-guanylyl cyclase from the aquatic fungus *Blastocladiella emersonii*. BAOJ Phys..

[53] Penzkofer A., Stierl M., Hegemann P., Kateriya S. (2011). Thermal protein unfolding in photo-activated adenylate cyclase nano-clusters from the amoeboflagellate *Naegleria gruberi* NEG-M strain. J. Photochem. Photobiol. A Chem..

[54] Weigand R., Rotermund F., Penzkofer A. (1997). Aggregation dependent absorption reduction of indocyanine green. J. Phys. Chem. A.

[55] Penzkofer A., Shirdel J., Zirak P., Breitkreuz H., Wolf E. (2007). Protein aggregation studied by forward light scattering and light transmission analysis. Chem. Phys..

[56] Holzer W., Pichlmaier M., Penzkofer A., Bradley D.D.C., Blau W.J. (1999). Fluorescence spectroscopic behavior of neat and blended polymer thin films. Chem. Phys..

[57] Penzkofer A. (2012). Photoluminescence behavior of riboflavin and lumiflavin in liquid solutions and solid films. Chem. Phys..

[58] Madge D., Wong R., Seybold P.G. (2002). Fluorescence quantum yields and their relation to lifetimes of rhodamine 6G and fluorescein in nine solvents: Improved absolute standards for quantum yields. Photochem. Photobiol..

[59] Birkmann C., Penzkofer A., Tsuboi T. (2003). Fluorescence excitation spectroscopic characterization of colour centres in a LiF crystal. Appl. Phys. B.

[60] Dörr F. (1966). Spectroscopy with polarized light. Angew. Chem. Int. Ed..

[61] Lindsey J. PhotochemCAD Spectra by Category. https://omlc.org/spectra/PhotochemCAD/html/.

[62] Tsuboi T., Penzkofer A., Lammel O. (2003). Oscillator strength of F_2_^-^ colour centres in LiF crystal. Opt. Quantum Electron..

[63] Strickler S.J., Berg R.A. (1962). Relationship between absorption intensity and fluorescence lifetime of molecules. J. Chem. Phys..

[64] Birks J.B., Dyson D.J. (1963). The relations between the fluorescence and absorption properties of organic molecules. Proc. R. Soc. Lond. Ser. A.

[65] Deshpande A.V., Beidoun A., Penzkofer A., Wagenblast G. (1990). Absorption and emission spectroscopic investigation of cyanovinyldiethylaniline dye vapors. Chem. Phys..

[66] Weigand R., Rotermund F., Penzkofer A. (1997). Degree of aggregation of indocyanine green in aqueous solutions determined by Mie scattering. Chem. Phys..

[67] Bohren G.F., Huffman D.R. (1983). Absorption and Scattering of Light by Small Particles.

[68] Gratz H., Penzkofer A., Weidner P. (1995). Nanometer particle size, pore size, and specific surface determination of colloidal suspensions and porous glasses by Rayleigh light scattering. J. Non Cryst. Solids.

[69] Fleming G.R. (1986). Chemical Applications in Ultrafast Spectroscopy.

[70] Voet D., Voet J.G. (2004). Biochemistry.

