# Supplementary material for: Absorption and Emission Spectroscopic Investigation of the Thermal Dynamics of the Archaerhodopsin 3 Based Fluorescent Voltage Sensor Archon2
Source: Int J Mol Sci. 2020 Sep 8;21(18):6576. doi: 10.3390/ijms21186576 (PMC7555599; doi:10.3390/ijms21186576)
Supplement: Supplementary file 1 [file ijms-21-06576-s001.zip › ijms-908500-supplementary.docx]

Article

Supplementary Materials: Absorption and Emission Spectroscopic Investigation of the Thermal Dynamics of the Archaerhodopsin 3 Based Fluorescent Voltage Sensor Archon2

Alfons Penzkofer 1,*, Arita Silapetere 2 and Peter Hegemann 2

S1. Amino Acid Sequence of Archon2

The amino acid sequence of Archon2 including the linker ENLYFQS LVDLEHHHHH H is displayed in Figure S1. The apoprotein molar mass is *M*apo = 29.58 kDa (without the linker the molar mass is 27.30 kDa). It contains 11 phenylalanine (Phe, F), 7 tryptophan (Trp,W), and 12 tyrosine (Tyr, Y) residues.

10 20 30 40 50 60
MDPIALQAGY DLLGDGRPET LWLGIGTLLM LIGTFYFLVR GWGVTDKDAR EYYAVPILVS
 70 80 90 100 110 120
GIASAAYLSM FFGIGLTEVP VGGEMLDIYY ARYAHWLFST PLLLLDLALL AKVDRVIIGT
 130 140 150 160 170 180
LVGVDALMIV TGLIGALSHT AIARYSWWLF STICMIVVLY VLATSLRSAA KERGPEVAST
 190 200 210 220 230 240
FNILTALVLV LWTAYPIIWI IGTEGAGVVG LGIETLLFMV LDVTCKVGFG FILLRSRAIL
 250 260 270
GDTEAPEPSA GADENLYFQS LVDLEHHHHH H

**Figure S1.** Amino acid sequence of Archon2 (= Arch T56P-P60S-T80P-D95H-T99S-T116I-F161V-T183I-L197I-A225C).

S2. Structural Formulae of Retinal Schiff Bases

In Figure S2 structural formulae of some protonated retinal Schiff base (PRSB) isomers and unprotonated retinal Schiff base (RSB) isomers are displayed.

|  | Protonated retinal Schiff base *all-trans,15-anti* |
| --- | --- |
|  | Protonated retinal Schiff base *13-cis,15-anti* |
|  | Retinal Schiff base *all-trans,15-anti* |
|  | Retinal Schiff base *13-cis,15-anti* |

**Figure S2.** Structural formulae of some protonated retinal Schiff base and neutral retinal Schiff base isomers (taken from [42]). The arrows indicate deviation from planarity.

S3. Determination of Absorption Cross-section Spectra of Ret_586 and Ret_380

The absorption cross-section spectra a(λ) are deriv ed from the absorption coefficient spectra αa(λ) by the relation

, (S1)

where *N* is the molecule number density. The absorption coefficient αa(λ) of Archon2 is composed of apoprotein and retinal contributions according to

. (S2)

The apoprotein absorption coefficient is given by

, (S3)

where *N*apoprotein is the apoprotein number density. The apoprotein absorption cross-section is composed of contributions from phenylalanine (Phe), tryptophan (Trp), and tyrosine (Tyr) residues according to

. (S4)

The Archon2 apoprotein contains 11 Phe (*n*Phe = 11), 7 Trp (*n*Trp = 7), and 12 Trp (*n*Tyr = 12). In Figure 1 at  = 280 nm it is αa(280 nm) = 2.76 cm-1. The retinal contribution is set to be αa,retinals(280 nm) = 0.31 cm-1 [42] giving αa,apoprotein(280 nm) = 2.45 cm-1 (Equation (S2)). The absorption cross-sections of Phe, Trp, and Tyr at 280 nm are σa,Phe(280 nm) = 3.8×10-21 cm2 [61], σa,Trp(280 nm) = 2.10×10-17 cm2 [61], and σa,Tyr(280 nm) = 4.62×10-18 cm2 [61] giving σa,apoprotein(280 nm) = 2.025×10-16 cm2 (Equation (S4)) and *N*apoprotein = 1.21×1016 cm-3 (Equation S3). The retinal number density *N*retinals is equal to the apoprotein number density (one retinal isomer is covalently bound to one opsin apoprotein) and is composed of the number density *N*Ret_586 of protonated retinal Schiff base Ret_586 and the number density *N*residual retinals of residual retinal components according to

. (S5)

The fraction residual retinals = *N*residual retinals/*N*Ret_586 is approximated by the ratio of the S0-S1 absorption coefficient integrals, αa,residual reinals,int and αa,Ret_586,int which are given by

, (S6)

and

. (S7)

( is wavenumber). Integrating αa,residual reinals(λ) and αa,Ret_586(λ) of Figure 1 results in αa,residual reinals,int = 1488.3 cm-2, αa,Ret_586,int = 5420.3 cm-2, and residual retinals = 0.275. The number density of Ret_586 is obtained from Equation (S5) to be

. (S8)

Insertion of values gives *N*Ret_586 = 9.49×1015 cm-3 and Ret_586 = (1+residual retinals)-1 = 0.784 (*N*apoprotein = *N*retinals = 1.21×1016 cm-3). The number density of residual retinals is

. (S9)

The obtained value is *N*residual retinals = 2.61×1015 cm-3.

The resulting absorption cross-section spectrum

(S10)

is displayed by the solid curve in Figure S3.

The S0-S1 absorption cross-section integral of Ret_586 is = 5.75×10-13 cm. Its oscillator strength is given by [62]

, (S11)

where *n* is the refractive index averaged over the S0-S1 absorption band of Ret_586 (here *n* ≈ *n*water ≈ 1.333). Insertion of parameters gives *f*Ret_586 = 0.546. The full spectral half-width of the S0-S1 absorption band is = 3400 cm-1.

**Figure S3.** Absorption cross-section spectra. Solid curve: Ret_586 (PRSB) of Archon2 in pH 8 Tris buffer (determined from Figure 1 and estimated Ret_586 number density). Dashed curve: Ret_380 (RSB, determined from heat degraded sample at  = 64.9 °C of Figure 4b).

The absorption cross-section spectrum of the deprotonated retinal Schiff base Ret_380 is shown by the dashed curve in Figure S3. It was determined from the absorption coefficient spectrum αa(λ, =64.9 °C) of Fig.4b by subtracting the residual retinal isomer contributions αa,residual retinals(=19.4 °C) as well as the remaining Ret_586 contribution αa,Ret_586(λ,=64.9 °C) and dividing by the number density of formed Ret_380 isomers *N*Ret_380(= 64.9 °C). The relations are

 (S12)

with



(S13)

and

, (S14)

where

, (S15)

, (S16)

. (S17)

The values are = 0.42, = 0.58, and = 1.013×1016 cm-3 (αa(586 nm, 19.4 °C) = 1.505 cm-1, a,Ret_586(586 nm) = 1.485×10-16 cm2).

The S0-S1 absorption cross-section integral of Ret_380 is = 5.95×10-13 cm. Its oscillator strength is

, (S18)

giving *f*Ret_380 = 0.56 (*n* ≈ *n*water ≈ 1.34). The full spectral half-width of the S0-S1 absorption band is = 6500 cm-1.

S4. Estimation of Radiative Lifetimes and Fluorescence Lifetimes of Ret_586 and Ret_380

The radiative lifetimes rad of Ret_586 and Ret_380 of Archon2 are determined from their S0-S1 absorption cross-section spectra a(λ) of Figure S3 and their average fluorescence wavelengths by using the Stricker-Berg relationship between absorption strength and radiative emission lifetime rad. The Strickler-Berg formula reads [63–65]

, (S19)

where *n*A and *n*F are the average refractive indices of the aqueous buffer solution in the S0–S1 absorption band region and the S1–S0 emission band region, respectively, and *c*0 is the velocity of light in vacuum. The average S1–S0 fluorescence emission wavelength is

, (S20)

and the absorption cross-section strength of the S0–S1 absorption band is

. (S21)

For Ret_586 we determine = 757 nm (top part of Figure 2), = 3.23×10-17 cm2 (Figure S3), *n*F = 1.33, *n*A = 1.3328 and calculate rad = 10.1 ns. For Ret_380 we use ≈ 460 nm, = 2.21×10-17 cm2 (Figure S3), *n*F = 1.3366, *n*A = 1.3406 and calculate rad = 3.31 ns.

Average Strickler-Berg based fluorescence lifetimes τF,SB are obtained from the radiative lifetimes and the fluorescence quantum yields according to

. (S22)

The obtained values are τF,SB(Ret_586) ≈ 106 ps (φF ≈ 0.0105) and τF,SB(Ret_380) ≈ 26.5 ps (φF ≈ 0.008).

S5. Normalized Fluorescence Excitation Quantum Distributions of a Fresh Thawed Archon2 Sample

In Figure S4 normalized fluorescence excitation quantum distributions of the fresh thawed Archon2 sample used in Figures 1 and 2 are displayed. Curves are shown for fluorescence detection wavelengths Fdet in the range from 300 nm to 720 nm. The normalization is made so that is equal to αa(λ=586 nm), i.e.

, (S23)

where is the un-normalized fluorescence excitation quantum distribution [59].

The curve represents the absorption coefficient spectrum of Ret_586. It is shown by the dashed curve in Figure 1. The normalized fluorescence excitation spectra for F,det = 620 nm to 380 nm in the fluorescence excitation wavelength region from F,exc = 500 nm to 310 nm are caused by residual retinal isomers present in the investigated fresh thawed Archon2 sample. The normalized fluorescence excitation spectra for F,det = 360 nm to 300 nm in the fluorescence excitation wavelength region from F,exc = 310 nm to 240 nm are caused by the Archon2 apoprotein mainly due to Trp absorption.

**Figure S4.** Normalized fluorescence excitation quantum distributions of a fresh thawed Archon2 sample in pH 8 Tris buffer for the fluorescence detection wavelengths F,det listed in the sub-figures. The thick dotted curves show the absorption coefficient spectrum αa(λ) of the investigated sample.

S6. Temporal Development of Attenuation Coefficient Spectra of Archon2 Stored at Room Temperature

The temporal development of the attenuation coefficient spectra α(λ) of Archon2 in pH 8 Tris buffer in the dark at room temperature is shown in Figure S5. The attenuation coefficient spectra α(λ) are composed of absorption coefficient contributions αa(λ) and scattering coefficient contributions αs(λ), i.e. α(λ) = αa(λ) + αs(λ). The main figure shows the attenuation coefficient spectra for storage times in the range from 0 to 18 days, and the inset shows the development of the attenuation coefficients α(800 nm), α(586 nm) and α(372 nm) versus storage time.

The curve α(800 nm,*t*storage) = αs(800 nm,*t*storage) shows the light scattering coefficient development with storage time in the transparency region of Archon2 at 800 nm caused by partial protein unfolding and associated aggregation [53–55,66–68]. During the first two days of sample storage no measurable light attenuation due to light scattering was observed (negligible protein unfolding and associated aggregation, αs(800 nm) ≈ 0). Then over the next two days (*t*storage = 2 d to 4 d) αs(800 nm) increased indicating the occurrence of some protein unfolding with associated aggregation. During the next four days some decrease of αs(800 nm) was observed likely due to less dense aggregate packing (decreasing monomer volume fill factor, larger aggregate size at the same degree of aggregation) with change-over to Mie scattering (Mie scattering is less efficient than Rayleigh scattering at the same degree of aggregation due to increasing destructive light interference with increasing aggregate size [55,66]). After 18 days of storage the light attenuation in the transparency region was largest likely due to continued partial protein unfolding with associated aggregation [53].

The decrease of light attenuation in the absorption region of Archon2 during the first 2 days of storage in the dark is thought to be caused by a decrease of the absorption coefficient spectrum αa(λ) of Archon2 because of some protein clustering to aggregates at condensation nuclei (specific surface area reduction of protein clusters compared to separated proteins [54], concentration of non-clustered normally absorbing proteins is reduced). For longer storage times the attenuation coefficient development in the absorption wavelength region is dominated by the light scattering coefficient contribution.

**Figure S5.** Temporal development of attenuation coefficient spectra α(λ) of Archon2 in pH 8 Tris buffer at room temperature ( = 21±1 °C) in the dark. The storage times are listed in the legend. The inset shows the development of the attenuation coefficients at pr = 800 nm (light scattering), 586 nm (Ret_586 absorption), and 372 nm (S0-Sn absorption of Ret_586 and absorption of residual retinal isomers).

S7. Lower Limit of Activation Barrier of Thermal Ground-State Isomerization

In the thermal aging studies of Archon2 in pH 8 Tris buffer at 3 °C over a period of 22 days and at 21 °C over a period of 18 days no indication of Ret_586 isomer conversion to other isomer forms was observed. This observation indicates a high isomerization activation barrier *E*act,Ret_586 which hinders isomer conversion within the applied storage times.

Taking the experimental data of Archon2 at room temperature we estimate in the following a lower limit of the activation barrier of ground-state isomerization. Since no isomerization is observed, the time constant of Ret_586 isomerization iso,Ret_586 has to be larger than about three times the maximum sample storage time *t*storage,max, i.e. iso,Ret_586 > 3×*t*storage,max. The time constant of Ret_586 isomerization is related to the corresponding energy activation barrier *E*act,Ret_586 by the Arrhenius relation [69] according to

, (S24)

where 0 = *h/*(*k*B) is the attempt time constant of barrier crossing [53,70] (*h* is the Planck constant, *k*B is the Boltzmann constant). Solving Equation (S24) for the activation energy barrier gives

. (S25)

Insertion of parameters (ϑ = 294 K, τ0 ≈ 1.6×10−13 s, iso,Ret_586 > 3×tstorage,max = 54 d) leads to *E*act,Ret_586 > 1.819 × 10−19 J = 9152 cm−1 × *hc*0.
